# Regular sling core stabilization training improves bone density based on calcium and vitamin D supplementation

**DOI:** 10.1186/s12891-023-06896-8

**Published:** 2023-10-13

**Authors:** Jing Liu, Daoming Xu, Lanying Liu, Chihuan Huang, Zhijie Guo, Di Zhang, Liyu Wei

**Affiliations:** 1 Affiliated Hospital of Nanjing University of Traditional Chinese Medicine, Nanjing, Jiangsu Province China; 2 Nanjing University of Traditional Chinese Medicine, Nanjing, Jiangsu Province China

**Keywords:** Primary osteoporosis, Bone density, Sling core stabilization training

## Abstract

**Background:**

Primary osteoporosis refers to a disease of aging characterized by reduced bone mass, damage to bone tissue microarchitecture, and predisposition to fracture.Sling core stabilization training emphasizes activating the deep local muscles of the spine under unstable conditions, and enhancing the body’s balance and control during exercise.

**Case presentation:**

A 70-year-old female went to the Acupuncture and Rehabilitation Department due to low back pain caused by osteoporosis.The patient received sling core stabilization training three times a week based on Calcium and Vitamin D Supplementation. After training, the patient’s back pain was significantly relieved and insisted one year. In the physical examination of bone mineral density, the results showed that the value of bone mineral density was better than last year.The patients adhered to sling core stabilization training and observed the changes of bone mineral density every year basis on calcium and vitamin D supplementation.

**Discussion:**

However, cases of calcium and vitamin D supplementation-based regular sling core stabilization training that improves bone density in osteoporosis patients have been rarely reported. Our group shared cases and analyzed possible mechanisms, hoping to provide reference for the prevention and treatment of primary osteoporosis.

## Background

Primary osteoporosis has been confirmed as a global clinical and public health problem characterized by loss of bone mass and deterioration of bone tissue architecture, with the concomitant increase of the risk of fracture [1]. The prevalence of osteoporosis among the elderly worldwide is 21.7%, including 35.3% in females and 12.5% in males [2]. Osteoporosis severely affects the quality of life of osteoporosis patients with pain, spinal deformation, high risk of fracture occurrence, as well as increased psychological burden [3]. Fragility fractures are considered as the most serious complication of osteoporosis. An estimated 9 million fragility fractures are diagnosed worldwide each year [4], and women aged over 50 have the risk of fracture of about 40–50% [5]. Non-pharmacological treatment of osteoporosis comprises lifestyle modification, exercise therapy, and traditional Chinese medicine [3].

Calcium and vitamin D supplements are the key nutrients recommended by the anti-osteoporosis guidelines to maintain bone health and prevent and treat osteoporosi [5]. Increasing bone density and bone strength is the ultimate goal of osteoporosis treatment. Exercise is the most simple and persistent program for prevention and treatment of osteoporosis, but no long-term follow-up study on exercise to improve bone mineral density under aging conditions has been found [6].

Sling core stabilization training refers to an exercise therapy to facilitate trunk neuromuscular control under unstable conditions [7]. By setting different training programs to adjust the difficulty of training, enhance the strength and endurance of trunk muscles, activate the overall motion control system, thus enhancing the stability, coordination and control of the spine, and persistently improving the back pain [8]. Research has confirmed that regular exercise can delay bone mineral loss. It is unknown to observe whether long-term regular suspension core stability training can improve bone mineral density and the time required to improve bone mineral density on the basis of calcium and vitamin D supplementation. The case study presented in the following is a clinical case in which our research found a 6-year increase in bone density due to calcium and vitamin D supplementation-based regular Sling core stabilization training.

## Patient information

### History

The patient was a female retired worker aged 70 years, with Han nationality and college education. Since October 2016, the patient went to the Acupuncture and Rehabilitation Department of Jiangsu Province Hospital of Chinese Medicine for treatment due to repeated attacks of back pain. In 2000, a fall caused a rib fracture on the right side of the patient. Moreover, in 2012, she went to the hospital for bilateral shoulder pain, and the recommended bone density showed lumbar spine T: -3.7 and femoral neck T: -2.2. In addition, she was diagnosed with osteoporosis. However, the patient did not pay attention to osteoporosis and did not receive anti-osteoporosis treatment. The left radius fracture of the patient was caused by a fall in 2014.

### Relevant past interventions with outcomes

The patient was on a zoledronic acid intravenous drip at the Jiangsu Provincial People’s Hospital once a year at 5 mg from 2015 to 2018. The detailed data of calcium and vitamin D supplementation are presented as follows: daily vitamin D supplementation of 0.5ug in 2015; 600 mg of calcium and 0.5ug of vitamin D in 2016; 600 mg of calcium and 0.5ug of vitamin D in 2017–2018. The type of Vitamin D is Calcitriol (1a,25-dihydroxycholecalciferol-1a, 25(OH)2 D),and the calcium is calcium carbonate.

### Diagnostic assessment

The patient’s back exhibited generalized dull pain, weakness, and chills. Specialized physical examination showed it’s normal in Superficial sensation, Deep sensation, Patellar tendon reflexes and Achilles tendon reflexes in both lower extremities. The muscle tone was grade 0, the pathological reflexes of both lower extremities were negative (Babinskin ,Chaddock,Oppenheim,Gordonsand,Hoffmann sign), the Straight leg raising test was negative, the muscle strength of both lower extremities was grade 5, the lumbar spine dorsal strength was grade 4, the lumbar spine anterior flexion range was limited, and the one-legged standing test was accompanied by trunk torsion. Based on the above evaluation results, the preliminary diagnosis was nonspecific low back pain and no imaging was performed. We assessed the level of low back pain in patients before and after treatment by using VAS (the visual analog score), assessed the degree of lumbar spine dysfunction by using the ODI (the Oswestry Disability Index) score and quality of life by using the SF-36 scale (the MOS item short from health survey).

### Therapeutic intervention

**Fig. 1:**
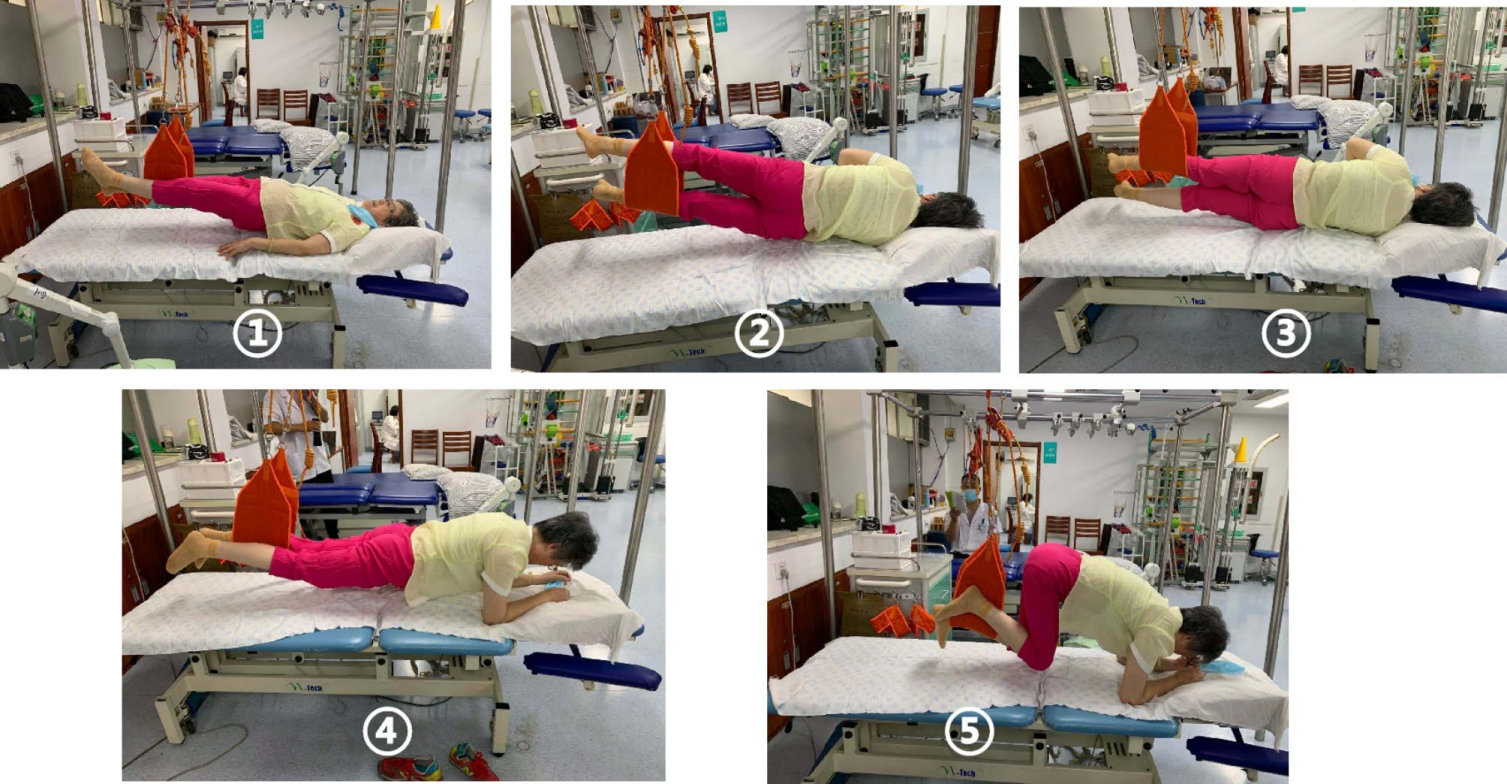
Sling core stabilization training. Action ①: Lie on the back with hands on both sides of your body. One side of the lower extremity is placed in a sling belt, the other side of the lower extremity is lifted to maintain the same height. Push down and lift the hips. Hold the position for 15 Sect. 5 times. (Bilateral alternation). Action ②: Lie on the side, the legs are placed in the sling, and the lower leg in the sling is forced to lift the pelvis, and the upper leg in the sling is also lifted.Hold the position for 15 sec 5 times. (Bilateral alternation). Action③: Lie on the side, one lower extremity placed in the sling belt with downward pressure, the other lower extremity lift to keep together. Hold the position for 10 Sect. 5 times. (Bilateral alternation). Action ④: Prone position, the upper limbs maintain 90° support position of shoulder and elbow. One lower limb is placed in the sling belt, and the other lower limb is stretched back to maintain the same height of both lower limbs. Push down the lower limbs located in the sling belt and lift the pelvis. Hold the position for 15 Sect. 5 times. (Bilateral alternation). Action⑤: Lift the pelvis with prone support after bilaterally flexing the lower extremities from the flexed hip to the extended hip and the extended knee 5 times. (Bilateral alternation)

**Sling core stabilization training**: The patient was trained 3 times per week from October 2016 to May 2018 and 2 times per week for 20–30 min from May 2018 to the present. (Figure 1).

### Follow-up and outcomes

#### Outcomes of low back pain

After 12 times of sling core stabilization training three times a week, the VAS score of the patient decreased from 4 to 2 points, and the ODI score also decreased from 26 to 10%, mainly reflected in the decrease of Pain Intensity, Personal Care, Lifting, Siting, Standing and Sleeping. The quality of life of the patient was improved due to the reduction of pain and the improvement of function. In the SF-36 scale, it showed the improvement of Physical Functioning, Role-physical, Bodily Pain, Social Functioning scores.We followed up on the patient’s low back pain after receiving the 2023 bone density report, and the results showed that regular sling core stabilization training based on calcium and vitamin D supplementation can prevent the occurrence of low back pain and maintain a good quality of life. (Tables 1 and 2) (Due to time reasons, the part of 2016 initial original evaluation data was lost, this is the original evaluation data from July to August 2017,We conducted a follow-up evaluation of low back pain in August 2023.)

**Table 1:** VAS and ODI results before and after treatment

	VAS	ODI										
		Total points	Pain Intensity	Personal Care	Lifting	Walking	Sitting	Standing	Sleeping	Sex Life	Social Life	Traveling
Before treatment	4	26%	2	1	1	0	3	3	3	0	0	0
After treatment	2	10%	1	0	1	0	2	1	0	0	0	0
Follow-up	1	4%	1	0	1	0	0	0	0	0	0	0

**Table 2:** SF-36 results before and after treatment

	SF−36							
	Physical Functioning	Role-physical	Bodily Pain	General Health	Vitality	Social Functioning	Role-Emotional	Mental Health
Before treatment	85	75	64	35	55	62.5	100	56
After treatment	95	100	94	37	45	75	100	56
Follow-up	95	100	100	62	65	87.5	100	76

### Outcomes of bone mineral density(BMD)

After treatment three times a week, the patients’ back pain was significantly reduced and their quality of life was significantly improved. Subjectively, the patient continued to carry out suspension core stability training three times a week. On May 23, 2017, the patients underwent annual bone mineral density re-examination, and the results showed that the bone mineral density showed an improvement trend. From May 2018, patients will reduce their training to 2 times a week. We continued to observe the annual bone mineral density report of patients until 2022, and found that on the basis of calcium and vitamin D supplementation, regular suspension core stability training improved bone mineral density. In 2023, Sling core stabilization training was suspended from January, 2023 to April, 2023 due to the liberalization of Covid-19 prevention and control in China and its infection. Calcium and Vitamin D are supplemented daily as normal. (Table 3)

**Table 3:** 2012–2023 Bone Mineral Density

	2012/7/3	2013/6/28	2014/8/7	2016/5/9	2017/5/23	2018/5/21	2020/5/20	2021/6/4	2022/6/8	2023/7/19
L1	-2.8	-2.8	-3.1	-3.0	-3.1	-2.8	-2.6	-2.8	-2.4	-2.8
L2	-3.9	-3.3	-3.6	-3.4	-3.6	-3.2	-3.1	-3.5	-3.4	-3.3
L3	-4.2	-3.4	-3.9	-3.9	-3.7	-3.5	-3.4	-2.7	-2.5	-2.9
L4	-3.7	-3.4	-3.3	-3.4	-3.3	-3.1	-2.8	-2.3	-2.0	-2.4
Lumbar Total	-3.7	-3.3	-3.5	-3.4	-3.5	-3.2	-3.0	-2.7	-2.5	-2.8
Femoral Neck(left)	-2.2	-2.1	-2.5	-2.3	-2.2	-2.3	-2.3	-2.5	-2.1	-2.2
Femoral Total (left)	-2.2	-2	-2.3	-2.1	-2.0	-2.0	-1.9	-1.6	-1.7	-1.7

**BMD testing**: From 2012 to 2023, the patient had 10 axial dual-energy X-ray absorptiometry (DXA) of the lumbar spine and left femoral neck. In 2015–2018, due to zoledronic acid treatment, BMD teating was in Jiangsu Province Hospital, and the rest were collected in Jiangsu Province Official Hospital. Unfortunately, the patient did not collect BMD in 2015 and 2019.

The patient did not receive any anti-osteoporosis treatment from 2012 to 2014. In July 2012, the first BMD examination was performed for the patient, whose BMD value met the diagnostic criteria for osteoporosis, but the patient refused anti-osteoporosis treatment and only accepted adjustments to daily lifestyle (avoidance of strong tea and caffeine intake, and increased outdoor activity time). In 2013 BMD values were better than 2012. In 2014 BMD results were worse than 2013 but better than 2012. Considering daily lifestyle adjustments could not improve bone mineral loss. Based on the changes in BMD in the previous three years, anti-osteoporosis medication was administered in 2015 on the advice of a physician. The 2017 and 2018 reports showed BMD values for the patient who received zoledronic acid combined with calcium and vitamin D supplementation for 2 and 3 years and sling core stabilization training 3 times per week for 2 years. 2020, 2021 and 2022 reports showed BMD values for patients with calcium and vitamin D supplementation and 3 consecutive years of 2 weekly sling core stabilization training. The reports indicated progressive improvement in both lumbar and hip BMD.The report of 2023 shows a decrease in BMD values in part lumbar and hip regions compared to 2022. Considering Covid-19 as the possible factor, suspension training will suspend for 3 months, and we will continue to observe.

## Discussion

When we alleviated low back pain through sling core stabilization training, we unexpectedly found that on the basis of calcium and vitamin D supplementation, the patient’s bone metabolism and bone density were improved, even in the 5 years after the patient stopped zoledronic acid. The improvement of bone mineral density of patients was analyzed as follows:

### The BMD decreased in osteoporosis patients is correlated with muscle

For anatomical location, bones provide attachment points for muscles, muscle wrapping protects bones, and muscle contraction produces limb movement. The likelihood of bony fractures as a result of falls is correlated with the bone’s increasing brittleness and the muscles’ diminished capacity to support the trunk and protect the bone. This patient had two fall-induced fractures before receiving sling core stabilization training, whereas no falls occurred after training. Thus, this result reveals that sling core stabilization training is capable of enhancing muscle stabilization control to improve bone protection.

From the functional perspective, muscle and bone interact as endocrine organs to secrete muscle-derived and bone-derived factors. Muscle secretes Myostatin, Irisin, and BAIBA for the regulation of bone metabolism [9]. Osteocalcin (OCN), wingless MMTV integration site family member 3a (Wnt3a), and insulin-like growth factor-1 (IGF-1) for the regulation of muscle function [10]. Furthermore, osteocytes sense the shear stress generated by bone fluid flow through dendrites and cell bodies while signaling to osteoblasts and osteoclasts to initiate bone remodeling [11]. BMD report showed that with aging, it is insufficient to improve bone mineral density in treatment with lifestyle modifications (2013 and 2014).Based on calcium and vitamin D supplementation, with sling core stabilization training for 2 years, patients showed a gradual improvement in BMD, which was even maintained until 5 years after zoledronic acid discontinuation. Accordingly, calcium and vitamin D supplementation-based long-term regular sling core stabilization training is capable of enhancing BMD in osteoporosis patients.

### Rationale for bone density enhancement by sling core stabilization training

Core muscles refers to a set of muscles with attachments to the spine, pelvis, and hips, supporting the spine and transmit force [12]. Bergmark divided the core muscles into local muscles and global muscles. Local muscles (e.g., transversus abdominis and multifidus) provide intersegmental stability of the spine; global muscles (e.g., erector spine, lumbar square, oblique abdominis, and rectus abdominis) control the direction of spinal activity [13]. Sling core stabilization training places stress on closed-chain exercise under unstable conditions to activate the control of movement by the nervous system, strengthen the feedback between nerves and muscle groups, improve the strength of deep stabilizing muscle groups in the spine, and improve the body’s balance and control during movement [14]. Existing research has confirmed that sling core stabilization training outperforms conventional training in increasing the strength and stability of spinal muscle groups [15]. A study by Touban BM [16] confirmed that the cross-sectional area of the core muscles of the lumbar abdomen is positively correlated with bone density. As revealed by the analysis of the trend of changes in BMD in this patient, calcium and vitamin D supplementation-based long-term regular sling core stabilization training increased the very cross-sectional area of the core muscles by increasing the very cross-sectional area of the core muscles to improve BMD.

### Calcium and vitamin D supplementation

Calcium and vitamin D supplementation also takes on a great significance to the prevention and treatment of osteoporosis. The patient in this case had a daily dose of calcium and vitamin D since 2015 when administrated with anti-osteoporosis medication. Calcium is critical to regulating muscle contraction, bone strength, nerve impulses, and conduction balance [17]. The Expert Consensus on Nutritional and Exercise Management in Patients with Primary Osteoporosis suggests that elderly Chinese osteoporosis patients should receive 500–600 mg of calcium and 400–600 IU of vitamin D daily [18]. Iacopo Chiodini’s analysis suggested that calcium and vitamin D supplementation increased BMD and reduced the risk of fracture [19]. However, the role of calcium and vitamin D supplementation in the treatment of osteoporosis is very limited, with calcium increasing BMD by only 1% in the first year of use and no further increase thereafter. Vitamin D did not increase BMD in clinical trials [20]. Analysis by Zhao JG et al. suggested that there is no significant relationship between calcium or vitamin D supplementation or both for osteoporotic fracture prevention [21]. Accordingly, it is inferred that the improvement in the BMD of the patient, in this case, was primarily attributed to 6 years of regular exercise training.

## Conclusion

There is currently a lack of multi-year follow-up on the effectiveness of anti-osteoporosis treatment, and medication sequential therapy is often preferred during treatments. The result of this report reveals that calcium and vitamin D supplementation-based long-term regular sling core stabilization training is capable of regulating bone metabolism and even increasing BMD since the combination of regular sling core stabilization training and calcium and vitamin D supplementation have consistently improved BMD values in this patient. Based on the bone mineral density report of 2017, the bone mineral density of patients showed an improvement trend after 8 months of suspension core stabilization training, and the bone mineral density of patients still improved after continuous regular training even after stopping anti-osteoporosis drugs.Therefore, the patient in this case’s existing anti-osteoporosis regimen may lay a basis for therapeutic therapy.

### Limitations

In the early stage, when receiving this case, only chronic low back pain was treated, and no intervention in osteoporosis was considered. On the basis of reducing pain, improving motor function and improving quality of life through suspension stability training, the patient made weekly training a part of life. Our research group found that the BMD of the patient driven by aging did not decrease after discontinuation of zoledronic acid, which provided a realistic basis for the prevention and treatment of osteoporosis by exercise. Unfortunately, we do not know the effect of sling core stabilization training on the bone metabolism index of the patient on the basis of calcium and vitamin D supplementation.

## Data Availability

The authors declare that all data used during the study appear in the submitted article.
